# Late developing mammary tumors and hyperplasia induced by a low-oncogenic variant of mouse mammary tumor virus (MMTV) express genes identical to those induced by canonical MMTV

**DOI:** 10.1186/1476-4598-12-79

**Published:** 2013-07-18

**Authors:** Robert D Bruno, Sonia M Rosenfield, Gilbert H Smith

**Affiliations:** 1 Mammary Stem Cell Biology, CCBB, CCR, NCI, Bethesda, MD 20892, USA

**Keywords:** Mammary tumorigenesis, Mouse mammary tumor virus, Nodule inducing virus, Mtv-1, Common integration sites

## Abstract

**Background:**

The canonical milk-transmitted mouse mammary tumor virus (MMTV) of C3H mice (C3H-MMTV) rapidly induces tumors in 90% of infected animals by 8 months of age. Pro-viral insertions of C3H-MMTV into genomic DNA results in the overexpression of common core insertion site (CIS) genes, including Wnt1/10b, Rspo2, and Fgf3. Conversely, infection by either the endogenous Mtv-1 virus (in C3Hf) or the exogenous nodule-inducing virus (NIV) (in Balb/c NIV) induces premalignant mammary lesions and tumors with reduced incidence and longer latency than C3H-MMTV. Here, we asked whether Mtv-1/NIV affected the expression of core CIS genes.

**Findings:**

We confirmed the presence of active virus in Mtv-1/NIV infected tissues and using quantitative reverse transcription PCR (qRT-PCR) found that Mtv-1/NIV induced neoplasms (tumors and hyperplasia) commonly expressed the core CIS genes Wnt1, Wnt10b, Rspo2, Fgf3.

**Conclusions:**

These results underscore the importance of core CIS gene expression in the early events leading to MMTV-induced mammary tumor initiation regardless of the viral variant.

## Findings

The exogenous mouse mammary tumor virus (MMTV) of C3H mice is transmitted exclusively through the milk from a mother to her offspring during lactation. Infected glands develop hyperplasia and tumors, with a 90% mammary tumor incidence by 8 months of age [1-3]. Foster nursing of C3H pups on mothers from a mouse strain lacking exogenous virus results in the C3Hf strain of mice. C3Hf mice developed tumors at a reduced frequency (20%–40%) and an increased latency (14 to 18 months) [3-5]. The causative agent of C3Hf neoplasms was identified as a genetically encoded virus, Mtv-1, also known as the nodule inducing virus (NIV) [5].

To study the biology of this low tumorigenic virus, Lawrence Young and colleagues developed the Balb/c NIV strain by infecting Balb/c mice with NIV through a complex mammary transplantation protocol [6]. The result was a milk-transmitted virus that, like the endogenous Mtv-1 in C3Hf mice, produced tumors at lower frequency and longer latency compared to C3H-MMTV induced tumors in Balb/c mice. Balb/c NIV induced mammary epithelial hyperplasia in 95% of aged multiparous tissues. Hyperplastic lesions isolated from Balb/c NIV and propagated by serial passage in epithelium-free mammary fat pads of syngeneic females showed mammary tumor incidences at reduced frequency and an increased latency compared to transplanted hyperplastic outgrowths resulting from C3H-MMTV infection (50% versus 100%, and 13.5 months versus 7.7 months, respectively) [6]. Restriction digest analysis of the proviral cDNA sequences of virus isolated from Balb/c NIV and C3Hf mice revealed differences between the two viruses’ long-terminal repeat (LTR) sequence; however, both viruses shared common genetic differences with C3H-MMTV [7].

Analysis of canonical, highly tumorigenic, MMTV insertions in genomic DNA of tumors and hyperplasia has identified common integration sites (CIS) [8]. CIS are regions containing MMTV insertions that affect a particular gene’s expression, and are found independently across multiple hyperplasia and tumors. As MMTV inserts randomly into the genome during DNA replication, CIS likely represent critical genetic cancer initiation events. CIS genes are often deregulated and/or mutated in human breast cancer samples [9]. Wnt1, Wnt10b, Rspo2, and Fgf3 have been identified as “core” CIS, as their up-regulation by MMTV insertions has been frequently identified in screens across various mouse and viral strains [8-11]. It should be noted that MMTV insertions can occur >100 kb from the effected gene, and the molecular mechanisms of their gene expression induction may be varied and complex [8-11]. So, although the precise location of the insertion may vary, the CIS can be verified by the effect of the insertion on gene expression.

**Figure 1:**
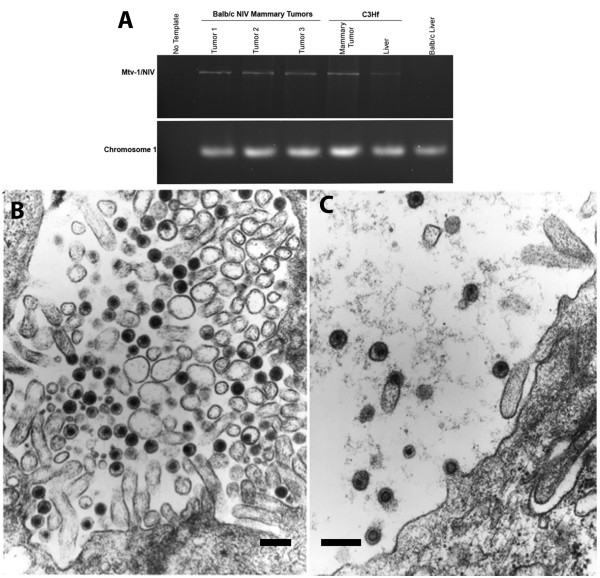
**Detection of the Mtv-1/NIV virus in Balb/c NIV mammary tumors. (A)** PCR amplification of a 3.0 Kb fragment from genomic DNA isolated from mammary tumors and liver tissues by Polymerase Chain Reaction (PCR) with MMTV (C3H)-specific primers. The MMTV (C3H) 3.0 Kb PCR product was detected in DNA isolated from Balb/c NIV mammary tumors, C3Hf mammary tumors and C3Hf liver tissue (positive controls) but not in Balb/c liver. Primers designed to the region 12918–1303 of Chromosome 1 were used as a loading control and to confirm genomic DNA quality. **(B** and **C)** Electron micrographs of ultrathin sections taken from mammary tumors of a C3H (MMTV) **(B)** and C3Hf (NIV) **(C)** mice show the presence of B-type retrovirions are abundant in both tumor types. Micrographs were taken of representative viral particles from ultrathin sections of OSO4 (osmium teroxide)-fixed tumor tissues, which had been stained with uranyl acetate and lead citrate to enhanced their electron density. The micrographs were taken on a Philips 300 electron microscope equipped with a camera. Scale bars = 200 nm.

To determine if the low oncogenic viruses Mtv-1/NIV affected these same core CIS genes, we analyzed mammary hyperplasia and tumors in C3Hf and Balb/c NIV mice. All mice were housed in Association and Accreditation of Laboratory Animal Care-accredited facilities in accordance with the NIH Guide for the Care and Use of Laboratory Animals. The National Cancer Institute Animal Care and Use Committee approved all experimental procedure. Breeding pairs of Balb/c NIV mice were a kind gift from Lawrence Young. From these pairs, we established a colony, and consistent with his findings, we identified tumors in only 39 out of 102 (38%) retired Balb/c NIV breeders, and never before the second year of life. From these mice, we transplanted suspected hyperplastic tissue and established 5 independent Balb/c NIV hyperplastic outgrowth (HOG) lines (Table 1). C3Hf tumors were collected from the Heston colony [3]. We confirmed the presence of virus by PCR with a primer set that identifies C3H-MMTV and Mtv-1/NIV, but not endogenous MMTV present within the Balb/c genome (Figure 1A) [12]. Genomic DNA samples isolated from Balb/c mammary tumors induced by MMTV from Czech mice, which is unrelated to C3H-MMTV, were also negative when tested with this primer set (data not shown). Ultrathin sections from mammary tumors of Balb/c NIV, C3Hf, and C3H mice were examined under an electron microscope. Intracellular nucleocapsids, budding forms, and extracellular retrovirions of the B type were found abundantly in both tumor types (Figure 1B and C). Comparison of the viral particles found in canonical milk-borne MMTV-infected C3H mammary tumors and those in tumors from C3Hf and Balb/c NIV and C3H-MMTV were indistinguishable.

**Table 1:** Incidence and latency of mammary tumors in 5 serially passaged premalignant Balb/c outgrowth lines

**HOG line**	**Passages (Number of outgrowths)**	**Number of tumors**	**Latency of tumor formation (mos)**	**Percent tumor formation**
1	6(24)	9	13.5–22	37.5%
2	6(24)	10	15–22	41.7%
3	23(102)	31	14–22	30.4%
4	11(44)	13	16–22	29.5%
5	8(32)	11	14.5–22	34.4%

**Figure 2:**
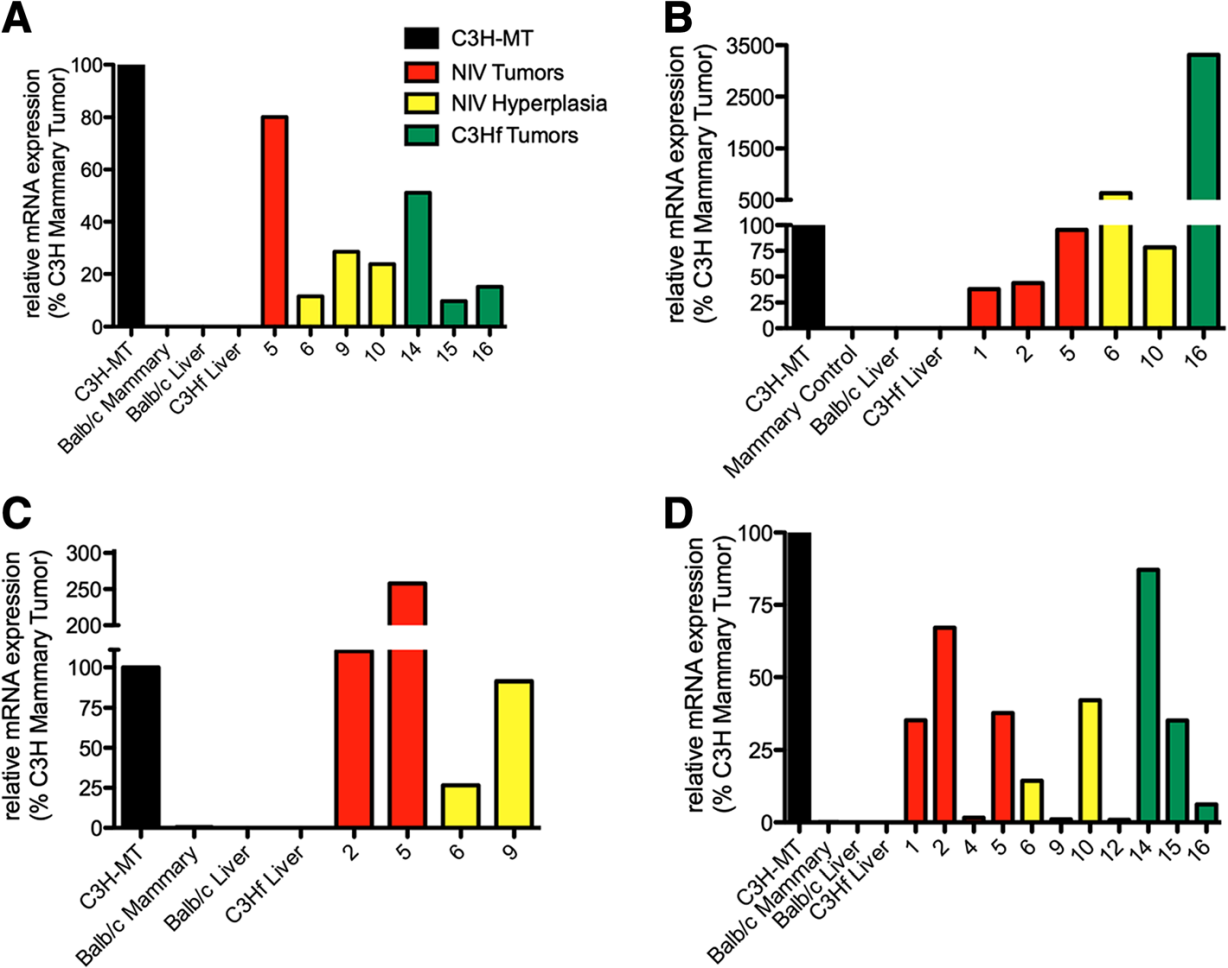
**Relative expression of core CIS genes in NIV/Mtv-1 induced tumors and hyperplasia.** The relative gene expression of Wnt1 **(A)**, Wnt10b **(B)**, Fgf3 **(C)**, and Rspo2 **(D)** in individual Balb/c NIV tumors (red), Balb/c NIV hyperplasia (yellow), and C3hf tumors (green) are plotted as a percentage of expression in a C3H tumor (black). mRNA levels were measured by qRT-PCR and expression was calculated using the 2^-ΔΔCt^ method. Only samples with significant expression levels above that detected in control mammary glands of Balb/c mice (Table 2) are shown.

Total RNA was isolated from spontaneous tumors and hyperplastic outgrowths (HOGs) for analysis by quantitative reverse transcription polymerase chain reaction (qRT-PCR) using validated primers to Wnt1, Wnt10b, Rspo2, and Fgf3 (SABiosciences/Qiagen, Valencia CA). Table 2 summarizes the samples analyzed, and in all, 5 Balb/c NIV tumors, 5 Balb/c NIV HOGs, and 6 C3Hf tumors were assayed. A C3H-MMTV mammary tumor (C3H-MT) was used as a positive control because of its high expression of all four genes. Importantly Wnt-1/10b, Rspo2, and Fgf3 are not expressed, or expressed at very low levels in normal mammary gland [8], and were not detected in pregnant mammary or liver control tissues from C3Hf and Balb/c NIV mice (Figure 2).

**Table 2:** Summary of significance* of expression of core CIS genes Fgf3, Rspo2, Wnt1, and Wnt10b

**Sample ID**	**Sample type**	**Fgf3**	**Wnt1**	**Wnt10b**	**Rspo2**
C3H-MT	MMTV (C3H) Mammary Tumor	+++	+++	+++	+++
1	Balb/c NIV Mammary Tumor	−	−	++	+++
2		++	−	++	+++
3		−	−	−	−
4		−	−	−	++
5		+++	+++	+++	+++
6	Balb/c NIV Hyperplastic Outgrowth (HOG)	+	++	+++	+++
7		−	−	−	−
8		−	−	−	−
9		++	++	−	+
10		−	++	+++	+++
11	C3Hf NIV Mammary Tumor	−	−	−	−
12		−	−	−	++
13		−	−	−	−
14		−	+++	−	+++
15		−	+	−	+++
16		−	+	+++	+++

^*^Significance of expression was determined by comparing ΔCt values of each sample with normal mammary controls by ANOVA with Dunnett’s post-hoc.

−not significant.

+ p < 0.05.

++ p < 0.01.

+++ p < 0.001.

However, we found significant expression (p < 0.05 by one-way analysis of variance (ANOVA) and Dunnett’s post-hoc comparison of 4 independent reactions of each sample to control pregnant mammary gland) of all four genes in C3Hf tumors, Balb/c NIV tumors, and Balb/c NIV hyperplasia (Tables 2 and 3). All but 5 samples had significant expression of at least one of the four CIS genes (Table 2). In all, 4/16 of the neoplasia expressed Fgf3, 7/16 expressed Wnt-1, 6/16 expressed Wnt10b, and 11/16 expressed Rspo2 (Table 3).

**Table 3:** Summary of the percentage of samples demonstrating significant* expression of Fgf3, Wnt1, Wnt10b, and Rspo2

**Sample type**	**Fgf3**	**Wnt1**	**Wnt10b**	**Rspo2**
Balb/c NIV Tumor	2/5	1/5	3/5	4/5
Balb/c NIV Hyperplasia	2/5	3/5	2/5	3/5
C3Hf NIV Tumor	0/6	3/6	1/6	4/6
Total	4/16	7/16	6/16	11/16

^*^ Significance of expression was determined by comparing ΔCt values of each sample with mammary controls by ANOVA with Dunnett’s post-hoc.

Relative expression of each transcript in every sample was calculated using the 2^-ΔΔCt^ method. Samples with expression levels significant above mammary controls are shown in Figure 2A-D, and plotted as a percentage of C3H-MT expression. The relative expression varies greatly from sample to sample, but samples with high levels of expression approaching, and in some cases greatly exceeding, that seen in C3H-MT, are present in all three sample sub-types (Balb/c NIV tumors, hyperplasia, and C3Hf tumor). While some samples showed significant expression above background, their expression level was only 1–2% of that seen in the high-expressing C3H-MT (e.g. sample 10 in Figure 2A, sample 3, 4, 9, 12, and 13 in Figure 2B). It should be noted, however, that C3H-MT was chosen for its high expression of all four genes. It does not represent the average expression seen in C3H-MMTV induced tumors, which can vary greatly in the level and amount of CORE CIS genes they express [8]. Furthermore, as these genes are not expressed in normal mammary tissues, even their relatively low expression may be of importance.

This is the first demonstration that the low tumorigenic NIV virus in both C3Hf and Balb/c NIV mice induces expression of Fgf3, Rspo2, Wnt1, and Wnt10b. These genes were previously identified as core CIS of canonical, high tumorigenic MMTV. The induction of the same genes by a different virus validates the interpretation of MMTV CIS studies, which indicated that the activation of these genes are important early initiation events in mammary tumorigenesis. Conversely, these results also demonstrate that while the inductions of Fgf3, Rspo2, and/or Wnt-1/10b are important initiation events, they are not sufficient to induce tumorigenesis. The difference in tumorigenic potential of NIV versus canonical MMTV is likely due to environmental tumor promoting factors. While the rate of infection and insertion may vary between the two viruses, this alone cannot account for the observations presented here as Balb/c NIV mice displayed longer latency in tumor development from hyperplastic tissue. New insertion events are not required for progression to tumorigenesis MMTV transformed tissues [8,9], and here we found that 3/5 HOGs analyzed had significant up-regulation of all four transcripts (Table 1 and Figure 2).

Earlier work indicated that infection with canonical MMTV resulted in an enhanced response of the host mammary tissue to exogenous prolactin [13-15]. This was not seen in C3H mice free of the milk-borne canonical MMTV. Our data suggests that the up-regulation of the core pathways is important for tumor initiation even in the absence of canonical MMTV, but progression to tumor requires promotional events that differ in mice infected with one or the other virus. Expression of the MMTV envelope protein in transgenic mice results in increased side branching and alveolar development in virgin mice [16]. Furthermore, mutations in the MMTV envelope protein immunoreceptor tyrosine-based activation motif (ITAM) attenuate MMTV-induced tumorigenesis but do not affect the insertion rate of the virus [16]. Type-specific immunologic differences among the envelope proteins of C3H-MMTV and NIV/Mtv-1 have been identified [17,18]; suggesting that variations in the viral envelope proteins may contribute to the differences in the latency of tumor development. Importantly, MMTV derived from Czech mice, which is a tumorigenic but unique variant of MMTV, has also been shown to affect the same core CIS [8,9]. Alternatively, differences in the expression levels of Core CIS genes may exist between mice infected with low oncogenic and high oncogenic viruses, however significant differences were not seen in the hyperplasia or tumors analyzed here with the single control C3H-MT tumor. Whatever factor(s) causes the difference in malignant progression, the important finding is that activation of CORE CIS genes occurs often in premalignant mammary lesions regardless of the MMTV variant, providing further evidence that the expression of these genes plays an important role in the early initiation of mammary oncogenesis.

The unique, low tumorigenic, NIV/Mtv-1 induces genes identified as core CIS of high tumorigenic MMTV, despite having a reduced tumorigenic potential. These results confirm the importance of Fgf3, Wnt-1/10b, and Rspo2 signaling in mammary tumor initiation, and the potential role of MMTV variants in tumor promotion.

## Abbreviations

MMTV: Mouse mammary tumor virus; C3H-MMTV: Canonical milk-borne MMTV of C3H mice; NIV: Nodule inducing virus; Mtv-1: Genetically encoded endogenous MMTV of C3Hf mice; CIS: Common integration sites; qRT-PCR: Quantitative reverse transcription polymerase chain reaction; ITAM: Tyrosine-based activation motif; ANOVA: One-way analysis of variance; MT: Mammary tumor; C3Hf: C3H mice foster nursed by mothers from a mouse strain lacking exogenous virus.

## Competing interests

The authors disclose no competing interests.

## Authors’ contributions

RDB participated in the design of the study and carried out the qRT-PCR experiments and analysis and drafted the manuscript. SMR carried out the PCR detection of MMTV and contributed to the drafting of the manuscript. GHS conceived the study, participated in its design, generated the EM images and contributed to the drafting of the manuscript. All authors read and approved the final manuscript.
